# Diagnostic delay for giant cell arteritis – a systematic review and meta-analysis

**DOI:** 10.1186/s12916-017-0871-z

**Published:** 2017-06-28

**Authors:** James A. Prior, Hoda Ranjbar, John Belcher, Sarah L. Mackie, Toby Helliwell, Jennifer Liddle, Christian D. Mallen

**Affiliations:** 1 Research Institute for Primary Care and Health Sciences, Keele University, ST5 5BG Newcastle, UK; 2 Leeds Institute of Rheumatic and Musculoskeletal Medicine, University of Leeds, Leeds, UK; 3 NIHR Leeds Musculoskeletal Biomedical Research Unit, Leeds, UK; 4 Institute of Health and Society, Newcastle University, Newcastle, UK

**Keywords:** Diagnostic delay, Giant cell arteritis, Meta-analysis, Systematic review

## Abstract

**Background:**

Giant cell arteritis (GCA), if untreated, can lead to blindness and stroke. The study’s objectives were to (1) determine a new evidence-based benchmark of the extent of diagnostic delay for GCA and (2) examine the role of GCA-specific characteristics on diagnostic delay.

**Methods:**

Medical literature databases were searched from inception to November 2015. Articles were included if reporting a time-period of diagnostic delay between onset of GCA symptoms and diagnosis. Two reviewers assessed the quality of the final articles and extracted data from these. Random-effects meta-analysis was used to pool the mean time-period (95% confidence interval (CI)) between GCA symptom onset and diagnosis, and the delay observed for GCA-specific characteristics. Heterogeneity was assessed by *I* ^2^ and by 95% prediction interval (PI).

**Results:**

Of 4128 articles initially identified, 16 provided data for meta-analysis. Mean diagnostic delay was 9.0 weeks (95% CI, 6.5 to 11.4) between symptom onset and GCA diagnosis (*I* ^2^ = 96.0%; *P* < 0.001; 95% PI, 0 to 19.2 weeks). Patients with a cranial presentation of GCA received a diagnosis after 7.7 (95% CI, 2.7 to 12.8) weeks (*I* ^2^ = 98.4%; *P* < 0.001; 95% PI, 0 to 27.6 weeks) and those with non-cranial GCA after 17.6 (95% CI, 9.7 to 25.5) weeks (*I* ^2^ = 96.6%; *P* < 0.001; 95% PI, 0 to 46.1 weeks).

**Conclusions:**

The mean delay from symptom onset to GCA diagnosis was 9 weeks, or longer when cranial symptoms were absent. Our research provides an evidence-based benchmark for diagnostic delay of GCA and supports the need for improved public awareness and fast-track diagnostic pathways.

**Electronic supplementary material:**

The online version of this article (doi:10.1186/s12916-017-0871-z) contains supplementary material, which is available to authorized users.

## Background

Giant cell arteritis (GCA) is the most common form of medium and large-vessel vasculitis [1]. Inflammation typically affects head and neck arteries, including the superficial temporal and posterior ciliary arteries [2]. Symptoms are caused by local vascular ischaemia often combined with cytokine-mediated features [3]. Symptoms may include headache, jaw claudication, transient visual loss, scalp tenderness, and limb claudication [4]. If GCA is untreated, permanent visual loss or stroke may ensue [5], other potential complications include aortic aneurysm, dissection and rupture [6].

In the UK, 10 people per 100,000 are reported to be affected by GCA [7], with women being three times more likely to be affected than men [8]. GCA occurs after age 50 and its incidence increases with age [7, 9], with a strong association with polymyalgia rheumatica (PMR). High-dose glucocorticoids are a highly effective treatment for GCA [10]. Early diagnosis and treatment are believed to be crucial since visual loss may occur in up to 15–20% of patients with GCA before treatment is commenced, while visual loss after the first 1–2 weeks of treatment is very rare [11].

Diagnosis of GCA in primary care remains difficult. Primary care physicians are faced with the frequently non-specific nature of many early symptoms of GCA, its relative rarity and a high prevalence of similar symptoms in the general consulting population [3, 12]. Delay to diagnosis is therefore not unusual [13, 14]. Delay may also occur as patients may not be aware of the significance of GCA symptoms, such as jaw claudication and temporal artery abnormality, and therefore do not seek healthcare promptly [15].

The importance of understanding the extent of diagnostic delay, and the reasons associated with delay, has been widely investigated by those seeking to improve care for patients with other conditions, including ischaemic heart disease and cancers [16, 17]. This has led to the development of public health interventions to raise awareness [18, 19]. For GCA, a secondary care ‘fast-track’ referral pathway, combined with GP education, reported a significant reduction in the number of patients experiencing permanent sight loss compared to those going through usual care. Though multifactorial, the reduction in diagnostic delays played a role in achieving this reduction in sight loss [20].

Our aim was to systematically review the existing literature reporting the extent of delay in receiving a GCA diagnosis. Our specific objectives were (1) to determine a new evidence-based benchmark of the extent of this delay by pooling the mean time-periods between GCA symptom onset and diagnosis of GCA and (2) to examine the role of GCA-specific characteristics on delay.

## Methods

A systematic review and meta-analysis of research literature was conducted. Medical bibliographic databases were searched to identify articles containing data on the mean time-period between the onset of GCA symptoms and GCA diagnosis. Meta-analysis was used to determine a pooled estimate of the time-period of diagnostic delay and analysed with regards to different GCA-specific characteristics.

### Data sources, searches and study selection

The article search was performed using bibliometric databases (MEDLINE, CINAHL, PsycInfo and ISI web of knowledge). Article inclusion criteria were (1) a population with GCA and (2) reporting a time-period of diagnostic delay between the onset of GCA symptoms and GCA diagnosis as an outcome. No restrictions were placed on language and authors were contacted to locate articles where necessary. Diagnosis of GCA could be defined by positive temporal artery biopsy, by American College of Rheumatology (ACR) 1990 criteria [21], or by a documented clinical diagnosis of GCA. Articles were excluded if patients did not have GCA or did not report diagnostic delay.

From the total number of articles identified through all searches, a single reviewer (HR) initially screened the articles by title. Two reviewers (HR & JAP) independently screened articles by their abstracts and then, upon consensus, the remaining articles were reviewed in full (JAP & CDM). Finally, the reference list of each included article was checked for further relevant articles by a single reviewer (JAP).

### Data extraction

Data were extracted from eligible articles by two reviewers (JAP & TH). The primary outcome of interest extracted from the final included articles was the mean time-period between onset of GCA symptoms and GCA diagnosis and the related estimate of variance. Other data extracted included lead author name, publication year, time-period between which patients were recruited or sampled from medical records, sample size, sex, age, country, healthcare setting, GCA-specific characteristic, method of GCA diagnosis, and how a delay in diagnosis had been defined. GCA-specific characteristics were examined within three categories, namely (1) commonly-reported GCA symptoms (polymyalgic symptoms, visual manifestation, visual loss, headache, jaw claudication and scalp tenderness); (2) subtype of GCA (cranial or non-cranial, presence or absence of PMR, positive or negative biopsy result); and (3) sample demographic (age, geographical location and sex).

### Quality assessment

Two reviewers (JAP & TH) assessed the quality of the final articles using a modified version of the Newcastle-Ottawa quality assessment scale for cohort studies. Though articles could be cross-sectional, case–control or cohort in design, several criteria were chosen from the cohort version of the Newcastle-Ottawa tool as this best represented the qualities required.

### Data synthesis

The primary outcome of interest was the mean number of weeks between symptom onset and GCA diagnosis, with an accompanying estimate of variation (standard deviation (SD)); however, several articles reported data in other formats. Where possible, the corresponding author was contacted and data requested in the required format. Where data were not provided, data were converted to allow direct comparisons between datasets. Data conversion could occur in three instances, depending on the originally reported format. Firstly, if delay was reported in days or months, these values were converted to weeks. Secondly, if an article had reported the variance around a mean using a low to high range, then this was converted to a SD (using a formula from Hozo et al. [22], low to high range data was used to generate an imputed SD [23]). Thirdly, the SD for each dataset was converted to a standard error (SD/√n) for use in the meta-analysis.

### Analysis

All articles included in the systematic review were initially examined using a narrative synthesis, comparing the characteristics of these articles. Random-effects meta-analysis was used to report a pooled mean number of weeks (95% confidence interval (CI)) between symptom onset and GCA diagnosis. This meta-analysis was presented as a forest plot, with heterogeneity initially assessed using the *I* ^2^ statistic and then using 95% prediction intervals (PI) as advocated by Riley et al. [24]; 95% PIs may be added to summary results from random-effects meta-analyses to illustrate heterogeneity of effects that may not be fully conveyed by the 95% CI. Where there is a wide distribution of effect estimates with little overlap in confidence intervals, 95% PI can highlight a range of effects at the individual level across study settings and can prove more useful in clinical practice than a summary *I* ^2^ value.

Because the SD required imputation for several articles, sensitivity analyses were performed, firstly examining only those articles which originally reported SD, secondly only those articles which required imputation of SD, and thirdly those restricting GCA definition to biopsy-positive cases only. Finally, the extent of delay relating to GCA-specific characteristics was reported, with random-effects meta-analysis being conducted where there were a sufficient number of articles to do so.

## Results

### Search results

**Fig. 1:**
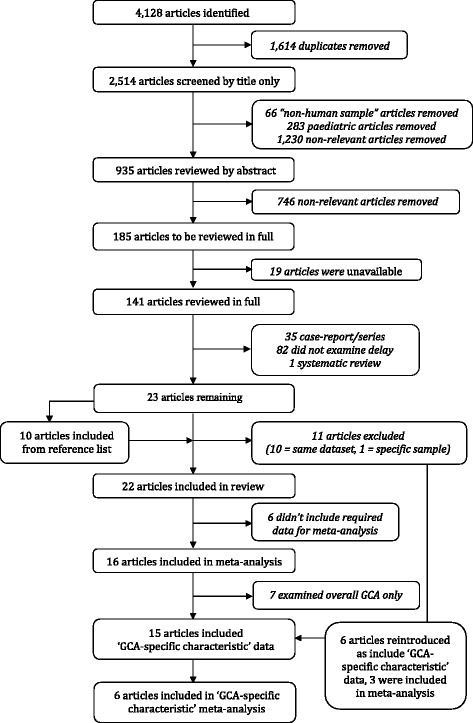
Selection of articles for inclusion in systematic review and meta-analysis

Out of 4128 articles initially identified, 141 were reviewed in full, leaving a total of 23 articles for inclusion. Of these, 11 were subsequently excluded as their datasets were duplicates of other articles. A further 10 additional articles were identified from reference lists. Therefore, 22 articles were included in the systematic review [11, 13, 20, 25–43], with 16 of these being pooled through meta-analysis [11, 13, 20, 26, 28, 30–33, 36, 37, 39–43]. From these 16 articles, 9 included GCA-specific characteristic data [11, 13, 28, 30–32, 37, 41, 43] and, when a further 6 previously excluded articles were reintroduced (articles using the same datasets now used in separate analyses), this totalled 15. Finally, 6 of these articles were included in the GCA-specific characteristic meta-analysis [11, 13, 28, 31, 41, 44] (Fig. 1).

### Sample characteristics

Of the 22 articles included in the systematic review, 10 came from England or the US. Two articles included patients from primary care and 16 had a retrospective study design. The 22 articles comprised 2474 GCA patients, of whom 72% were female and the average age was 73 years (mean ages ranging from 63–79, excluding the outlier of Hu et al. [34], which was removed due to a far younger mean age (43 years) and predominantly male sample (15:1 ratio of males to females)). A total of 17 articles defined GCA by a positive temporal artery biopsy, with the remainder using clinical diagnosis or ACR criteria. None of the included articles had examined diagnostic delay of GCA as their primary question; there was little information on how delay data was collected (Table 1).

**Table 1:** Characteristics of articles reporting delay of giant cell arteritis (GCA) diagnosis

Lead author, reference	Year	Sampling period	Country	Healthcare setting	Study design	Definition of delay in GCA diagnosis
Systematic review (n = 22)						
Calamia [25]	1981	1976–1978	USA	Tertiary care	Retrospective	Duration symptoms were present before diagnosis
Bella Cueto^a^ [26]	1985	1968–1983	Spain	Secondary care	Retrospective	Duration of symptoms until diagnosis
Karanjia [27]	1989	–	USA	Secondary care	Retrospective	Average delay from onset of symptom to biopsy
Desmet^a^ [28]	1990	1982–1988	Belgium	Secondary care	Retrospective	Time delay between presentation and biopsy
Kelkel [29]	1991	1984–1990	Switzerland	Secondary care	Retrospective	From first signs or symptoms to beginning of treatment
Myklebust^a^ [30]	1996	1987–1994	Norway	Secondary care	Prospective	Delay of diagnosis
Brack^a^ [31]	1999	1960–1996	USA	Tertiary care	Retrospective	Time to diagnosis
Duhaut^a^ [32]	1999	1991–1997	France	Secondary care	Prospective	Time interval between onset of symptoms and diagnosis
Nesher^a^ [33]	1999	1980–1995	Israel	Secondary care	Retrospective	Time from onset of symptoms to diagnosis
Hu [34]	2002	1999–2001	China	Secondary care	Prospective	Duration of symptoms before biopsy
Liozon^a^ [11]	2003	1977–2002	France	Secondary care	Retrospective	Delay in diagnosis
Nuenninghoff [35]	2003	1950–1999	USA	Primary & secondary	Retrospective	Time from onset of symptoms to GCA diagnosis
Gonzalez-Gay^a^ [36]	2004	1981–2001	Spain	Secondary care	Retrospective	Delay to diagnosis
Pease^a^ [37]	2005	–	England	Secondary care	Prospective	Time to presentation
Loddenkemper [38]	2007	–	England	Secondary care	Retrospective	Onset of symptoms prior to admission
Mari^a^ [39]	2009	1989–2007	Spain	Secondary care	Retrospective	Delay in diagnosis
Ezeonyeji^a^ [13]	2011	2003–2008	England	Secondary care	Retrospective	Symptoms onset to diagnosis
Mackie^a^ [40]	2011	2005–2009	England	Secondary care	Retrospective	Time between first onset of symptoms and first steroid treatment
Czihal^a^ [41]	2012	2002–2010	Germany	Secondary care	Retrospective	Time to diagnosis
Prieto-Gonzalez^a^ [42]	2012	2006–2011	Spain	Secondary care	Prospective	Duration of symptoms until diagnosis
Patil^a^ [20]	2015	2009–2013	England	Secondary care	Prospective	Duration of symptom until diagnosis
Singh^a^ [43]	2015	1950–2004	USA	Primary & secondary	Retrospective	Duration of symptoms
Initially excluded articles, subsequently used for characteristic-specific analysis only (n = 6)						
Gonzalez-Gay^b^ [45]	2000	1981–1998	Spain	Secondary care	Retrospective	Delay to diagnosis
Schmidt^b^ [46]	2000	1996–1999	Germany	Secondary care	Retrospective	Delay in therapy
Gonzalez-Gay [47]	2001	1981–1998	Spain	Secondary care	Retrospective	Delay to diagnosis
Gonzalez-Gay [48]	2003	1981–2001	Spain	Secondary care	Retrospective	Delay to diagnosis
Gonzalez-Gay^b^ [44]	2005	1981–2004	Spain	Secondary care	Retrospective	Delay to diagnosis
Lopez-Diaz [49]	2008	1981–2006	Spain	Secondary care	Retrospective	Delay to diagnosis

^a^Included in delay meta-analyses

^b^Included in characteristic-specific delay meta-analysis

### Diagnostic delay of GCA

The mean delay in receiving a diagnosis of GCA ranged from 1.2 (SD 1.6) to 34.7 (34.2) weeks. The majority of mean values had wide data ranges reported alongside them, with these often being skewed toward the higher value (Table 2). Five articles did not include all necessary data related to delay [25, 27, 29, 35, 38] and that of Hu et al. [34] was excluded (Additional file 1: Table S1), leaving 16 articles included in the meta-analysis [11, 13, 20, 26, 28, 30–33, 36, 37, 39–43].

**Table 2:** Extent of diagnostic delay reported within articles included in systematic review (*n* = 22)

			Sex	Age			Reported diagnostic delay				Converted diagnostic delay^a^	
Lead author, reference	Definition of GCA	*n*	% F	Mean	SD	Range	Time	Mean	SD	Range	Mean weeks of delay	SD
Calamia [25]	Positive TAB for GCA after fever was initial symptom	15	66.7	67	–	57–75	M	3^b^	–	–	–	–
Bella Cueto [26]	Positive TAB for GCA^c^	100	53	71.6	–	52–88	D	126	–	6–2190	18	52
Karanjia [27]	Positive TAB for GCA and/or study defined clinical criteria	63	–	–	–	–	D	52	–	–	–	–
Desmet [28]	Positive TAB for GCA	34	73.5	70.8	–	60–99	–	–	–	–	–	–
	Positive TAB for GCA, with cranial or polymyalgia symptoms^c^	21	–	–	–	–	D	8.5	10.9	1–40	1.2	1.6
	Positive TAB for GCA with constitutional (fever, fatigue, anorexia or weight loss) symptoms	13	–	–	–	–	D	21.5	27.9	2–105	3.1	4.0
Kelkel [29]	Positive TAB for GCA and/or study defined clinical criteria	130	74.6	76	7.5	60–92	M	5^b^	–	0.5–48	–	–
Myklebust [30]	Positive TAB for GCA, without PMR^c^	39	–	70.4	–	–	M	1.5	–	0.25–7.0	6.4	7.2
	Positive TAB for GCA, with PMR	15	–	74.4	–	–	M	1.9	–	0.5–5	8.1	10.7
Brack [31]	Large-vessel GCA (GCA diagnosis with vasculitic involvement)	74	88	66	–	52–85	M	8.1	–	0.1–48.0	34.7	34.2
	Cranial GCA (Positive TAB)^c^	74	78	72	–	54–89	M	2.6	–	0.5–11.0	11.1	7.5
Duhaut [32]	Incident cases of GCA, who satisfy inclusion criteria, including positive TAB^c^	207	75.8	75.6 (F) 74.1 (M)	8 7.4	– –	D	48	–	5–2113	6.9	50.2
	Incident cases of GCA, who satisfy inclusion criteria, with negative TAB	85	65.9	75.1 (F) 74.0 (M)	7.8 8.6	– –	D	33	–	4 – 1096	4.7	26.0
Nesher [33]	GCA defined using 1990 ACR criteria^c^	144	64.6	73.0	–	–	M	1.5	–	0.1–12	6.4	7.9
Hu [34]	Positive TAB for GCA or on clinical grounds (response to steroids)	16	6.3	43.1	–	28–60	M	5.5	–	0.25–24.3	–	–
Liozon [11]	Positive TAB for GCA^c^	175	64.6	75.2	7.1	–	D	79	83.5	–	11.3	11.9
	Silent GCA (constitutional symptoms, raised erythrocyte sedimentation rate)	21	66.7	74.3	7.9	–	D	123	–	30 – 360	17.6	7.9
	Overt cranial GCA	130	63.8	75.6	6.9	–	D	70	–	4 – 350	10.0	8.2
Nuenninghoff [35]	GCA defined using 1990 ACR criteria	168	79.2	75.6	–	–	D	40^b^	–	21–89	–	–
Gonzalez-Gay [36]	Positive TAB for GCA, with vascular involvement^c^	199	52.8	74.6	7.0	–	W	9.8	10.8	–	9.8	10.8
	Positive TAB for GCA, without vascular involvement	11	72.7	73.8	5.3	–	W	20.2	17.6	–	20.2	17.6
Pease [37]	GCA diagnosis (1990 ACR criteria) after initial presentation with polymyalgia symptoms^c^	42	–	71	–	60–81	M	3.0	–	0.4 – 22.1	12.9	23.3
Loddenkemper [38]	Positive TAB for GCA	90	74.4	74.6	7.8	–	D	125^b^	–	2–2555	–	–
Mari [39]	Positive TAB for GCA and 3 or more 1990 ACR criteria^c^	79	77.2	74.8	–	59–89	D	92	–	12 – 498	13.1	11.6
Ezeonyeji [13]	GCA in medical records^c^	65	72.3	75			D	35	–	2 – 336	5.0	11.9
Mackie [40]	GCA with ischaemic manifestation (GCA defined by 1990 ACR criteria, positive TAB or clinical features)^c^	222	71.0	72	–	67–78	D	64	98.3	12.3–78.5	9.1	14.0
Czihal [41]	GCA in medical records^c^	110	76.4	69	8.4	–	W	18.2	21.8	–	18.2	21.8
	Extra-cranial GCA	59	83.1	62.5	7.6	–	W	28.7	25.1	–	28.7	25.1
	Cranial GCA	51	68.6	73.7	7.0	–	W	6.5	6.6	–	6.5	6.6
Prieto-Gonzalez [42]	Positive TAB for newly-diagnosed GCA and 1990 ACR classification^c^	40	67.5	79.0	–	57–92	D	74.2	90.5	5 – 365	10.6	12.9
Patil [20]	Conventional pathway – GCA diagnosis in medical records^c^	46	71.7	75.4	7.6	–	D	32.0	39.5	1–196	4.6	5.6
	Fast-track pathway – GCA based on clinical features, lab results, biopsy and response to steroids	67	77.6	74.1	7.6	–	D	35.9	47.6	0–206	5.1	6.8
	GCA patients (total)^c^	204	79.9	76.0	8.2	–	D	41.3	95.5	–	5.9	13.6
Singh [43]	GCA without visual manifestation. Positive TAB for newly-diagnosed GCA and 1990 ACR classification	157	81.0	75.6	7.8	–	D	44.5	107.3	–	6.4	15.3
	GCA with visual manifestation. Positive TAB for newly-diagnosed GCA and 1990 ACR classification	47	77.0	77.4	9.2	–	D	30.6	31.1	–	4.4	4.4

^a^If the extent of diagnostic delay was reported as ‘days’ or ‘months’, this was converted to weeks

^b^Median

^c^Dataset used in meta-analysis

*ACR* American College of Rheumatology, *GCA* giant cell arteritis, *TAB* temporal artery biopsy, *PMR* polymyalgia rheumatica

Time: *D* days, *W* weeks, *M* months

**Fig. 2:**
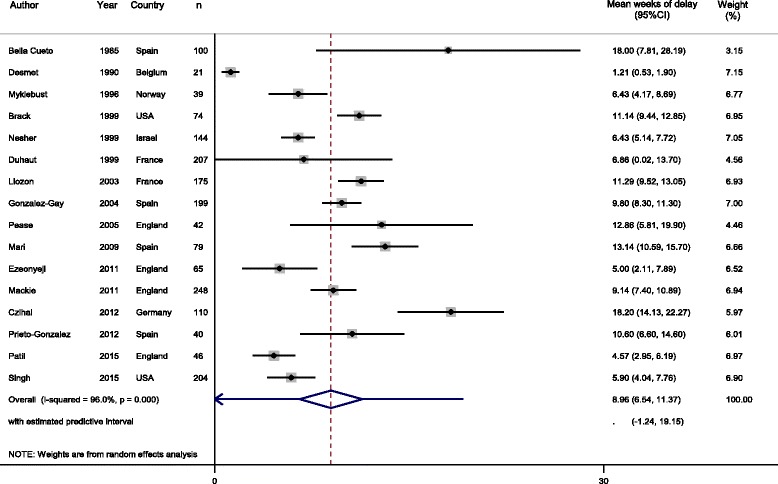
Meta-analysis of time-periods of delay in receiving a diagnosis of giant cell arteritis

The pooled mean time between GCA symptom onset and GCA diagnosis was 9.0 weeks (95% CI, 6.5 to 11.4) (*I* ^2^ = 96.0%, *P* < 0.001) (Fig. 2). Sensitivity analysis showed minimal difference in the length of delay if only articles that reported the original SD (8.7 (5.1 to 12.3) weeks, *I* ^2^ = 97.5%, *P* ≤ 0.001) (Additional file 1: Figure S1), imputed SD (9.1 (6.6 to 11.6) weeks, *I* ^2^ = 84.6%, *P* ≤ 0.001) (Additional file 1: Figure S2), or those that had defined GCA through temporal artery biopsy (8.6 (5.6 to 11.5) weeks, *I* ^2^ = 96.7%; *P* ≤ 0.001) (Additional file 1: Figure S3) were included.

### GCA-specific characteristic diagnostic delay

Nine articles included in the original meta-analysis also reported diagnostic delay for a particular GCA characteristic (Table 3). Six further articles [44–49] were reintroduced, their examination of GCA-specific characteristics meaning they could subsequently be compared against different datasets (Additional file 1: Table S2).

**Table 3:** Delay of giant cell arteritis (GCA) diagnosis by GCA-specific characteristic

			Mean delay by category				
Characteristics	Author	Year	*n*	Weeks (SD)	*n*	Weeks (SD)	*P* value^a^
Symptoms							
PMR				With		Without	
	Pease [37]	2005	42	12.9 (23.3)	–	–	–
	Ezeonyeji [13]	2011	14	6.0 (1.8)	–	–	–
Visual manifestation				With		Without	
	Gonzalez-Gay [45]	2000	42	9.6 (11.3)	119	11.5 (12.5)	0.19
	Ezeonyeji [13]	2011	23	3.0 (2.9)	–	–	–
	Singh [43]	2015	47	4.4 (4.4)	157	6.4 (15.3)	–
Visual loss				With		Without	
	Gonzalez-Gay [45]	2000	24	10.8 (13.6)	137	11.0 (12.1)	0.48
	Schmidt [46]	2000	5	7 (3)	–	–	–
	Ezeonyeji [13]	2011	16	1.7 (1.4)	–	–	–
Headache				Yes		No	
	Gonzalez-Gay [44]	2005	203	9.2 (9.9)	37	16.6 (15.0)	<0.001
	Ezeonyeji [13]	2011	54	4.3 (3.9)	–	–	–
Jaw claudication				Yes		No	
	Ezeonyeji [13]	2011	31	4.6 (2.8)	–	–	–
Scalp tenderness				Yes		No	
	Ezeonyeji [13]	2011	27	4.0 (2.9)	–	–	–
GCA							
Cranial vs. non-cranial				Cranial		Non–cranial	
	Desmet [28]	1990	21	1.2 (1.6)	13	3.1 (4.0)	<0.05
	Brack [31]	1999	74	11.1 (7.5)	74	34.7 (34.2)	<0.001
	Liozon [11]	2003	130	10.0 (8.2)	21	17.6 (7.9)	0.003
	Gonzalez-Gay [44]	2005	199	9.8 (10.8)	11	20.2 (17.6)	0.003
	Ezeonyeji [13]	2011	–	–	21	5.4 (3.5)	–
	Czihal [41]	2012	51	6.5 (6.6)	59	28.7 (25.1)	<0.01
GCA with PMR				GCA		GCA & PMR	
	Myklebust [30]	1996	39	6.4 (7.2)	15	8.1 (10.7)	–
	Gonzalez-Gay [44]	2005	144	8.3 (10.0)	96	13.4 (12.2)	<0.001
Biopsy result				Positive		Negative	
	Duhaut [32]	1999	207	6.9 (50.2)	85	4.7 (26.0)	–
	Gonzalez-Gay [47]	2001	161	7 (1.7)	29	8 (4.0)	0.6
Demographic							
Age				<69 years		≥70 years	
	Lopez-Diaz [49]	2008	46	13.2 (12.8)	227	9.4 (10.2)	0.03
Location				Rural		Urban	
	Gonzalez-Gay [48]	2003	132	9.9 (11.7)	78	11.1 (10.9)	0.23
Sex				Men		Women	
	Gonzalez-Gay [48]	2003	97	9.7 (12.6)	113	11.0 (10.4)	0.20

^a^Statistical comparison of groups from original article

*PMR* polymyalgia rheumatica

**Fig. 3:**
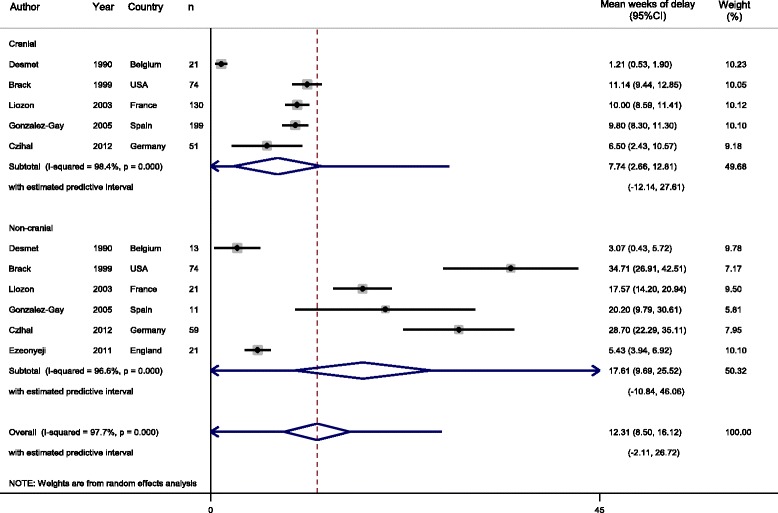
Meta-analysis comparing delay in diagnosis between GCA with cranial or non-cranial characteristics

Five articles had specifically compared diagnostic delay for those with cranial versus non-cranial GCA. Cranial GCA was defined as presentation with cranial features (e.g. headache, scalp tenderness) or positive temporal artery biopsy. Non-cranial delay was defined as presentation of GCA with constitutional symptoms (e.g. fever, anorexia or polymyalgia) or other non-cranial presentation. Each included article had originally reported a significantly greater delay in those with non-cranial GCA compared with cranial GCA. Our meta-analysis demonstrated that those with cranial GCA received a diagnosis after 7.7 weeks (2.7 to 12.8, *I* ^2^ = 98.4%, *P* < 0.001) and those with non-cranial GCA after 17.6 weeks (9.7 to 25.5, *I* ^2^ = 96.6%, *P* < 0.001) (Fig. 3).

No other characteristic had been reported often enough, included an appropriate comparator group or were from a unique dataset to allow further meta-analysis. However, within the original articles, significantly greater periods of delay had been reported in GCA patients without symptoms of headache compared to those with headache (16.6 vs. 9.2 weeks respectively, *P* ≤ 0.001) [44], for those with GCA and PMR compared to GCA only (13.4 vs. 8.3 weeks, *P* ≤ 0.001) [44], and for patients aged ≤ 69 years compared to those aged ≥ 70 (13.2 vs. 9.4 weeks, *P* = 0.03) [49].

Additionally, 95% PIs were calculated for each meta-analysis demonstrating an interval of 0 to 19.2 weeks for the mean time between symptom onset and GCA diagnosis (Fig. 2), 0 to 21.8 weeks for articles which reported SD only (Additional file 1: Figure S1), 1.0 to 17.2 weeks for those with imputed SD (Additional file 1: Figure S2), 0 to 20.2 weeks for articles where GCA had been defined through temporal artery biopsy (Additional file 1: Figure S3), 0 to 27.6 weeks for those with cranial symptoms (Fig. 3), and 0 to 46.1 weeks for those with non-cranial symptoms (Fig. 3).

### Quality appraisal

All articles included in the systematic review described samples broadly representative of GCA, based on age and sex distribution (except for Schmidt et al. [46]) and had ascertained the method of GCA diagnosis (typically temporal artery biopsy) from medical records (except for Pease et al. [37]). The majority of articles determined the time-period of diagnostic delay through review of medical records, as use of a retrospective cohort design was typical (Additional file 1: Table S3). Articles included in this review reported good quality of design, though little indication was provided on how delay was actually defined.

## Discussion

This systematic review and meta-analysis examined the extent of delay between first experiencing symptoms related to GCA and receiving a confirmatory GCA diagnosis, finding the mean time-period of diagnostic delay to be 9 weeks. Also of interest was how diagnostic delay is influenced by GCA-specific characteristics. Here, we found that even when patients present with distinct cranial symptoms, the delay in finally receiving a GCA diagnosis remains substantial (8 weeks) and is longer still for those with non-cranial symptoms (18 weeks). Such findings are of concern, as previous research has reported that as few as half of GCA patients can experience temporal headaches [3].

Achieving a prompt and accurate diagnosis of GCA remains challenging, demonstrated by typically wide and skewed time-periods of delay within individual studies. It was not uncommon for time-periods of delay to range from a single day in one patient, to a year in another from the same study. Further research is needed to fully describe the characteristics of patients experiencing both short and long periods of delay. When a patient presents to the clinician with mainly constitutional symptoms, such as fever or malaise, diagnosis is more challenging as these symptoms are common and frequently occur in other, more prevalent disorders. However, patients who present with classic cranial GCA or typically associated symptoms (e.g. headache, PMR) still experience a prolonged period of diagnostic delay, highlighting the need for an increased awareness of all facets of this condition.

Diagnostic delay is a common problem in many conditions. For example, a median 9-week delay has been identified in diagnosing childhood brain tumours [50], and a 24-week median delay in rheumatoid arthritis (RA) [51]. As the delay in receiving a diagnosis for such conditions has been shown to have negative effects on outcomes, much research has looked to reduce this respective diagnostic delay. It remains unclear at what point(s) in the patient pathway the greatest potentially avoidable delay is incurred [52]. Raza et al. [51] examined the reasons for delay in assessment of RA across Europe. They found that delays in receiving a RA diagnosis could be related to the time taken for (1) the patient to consult healthcare after symptom onset, (2) the patient to be given an appointment, (3) the primary care clinician to refer the patients to secondary care, and (4) the patient to receive a secondary care appointment; the extent of delay at each point varied across countries. There may also be more specific reasons for delay, for example, varying test availability (i.e. ultrasonography) due to different service provision by geographical region or local funding allocation. Linked to variations in the point at which delay occurs, the terminology of delay should also be reconsidered. Future research should make the distinction between ‘consultation delay’ (the period from symptom onset to receiving a consultation) and ‘diagnostic delay’ (the time between first consultation and final diagnosis). This acknowledges that clinical diagnosis is not possible until the patient initiates contact with a health professional. Research has demonstrated that through disease awareness programmes it is possible to reduce delay at any stage of the disease pathway [19] and thus the importance of our review exists in determining an evidence-based baseline level of delay in GCA diagnosis that future studies must attempt to reduce.

The strength of this systematic review and meta-analysis is that it provides the first systematic approach to pool diagnostic delay of GCA in the world literature. We have also collated those articles that have examined delay related to specific GCA characteristics to identify barriers to receiving a prompt diagnosis.

The primary limitation of our research is that heterogeneity may have been introduced due to the way in which delay data were recorded. In each article, delay was a secondary outcome and little (or no) information was provided on how this information was obtained, for example, as part of routinely recorded clinical care (either contemporaneously or retrospectively) or whether patients were asked as part of the study protocol. However, as the majority of articles did define delay through the same phrasing (the time between GCA symptom onset and GCA diagnosis), the manner in which this was collected may be less important. Furthermore, though more detail on the mechanisms of delayed GCA diagnosis would be of great benefit, from the perspective of the patient or clinician, this is the best data that we presently have to understand the current issue of delay and therefore provides our best estimate to date.

Several articles report diagnostic delay data which is skewed. Though this may be considered as an influence on our final pooled values, standard meta-analytic methods assume normality in the distribution of the means (but not the raw data) and they are valid when sample sizes within individual studies are sufficient to enable the central limit theorem to hold. Related to the variance observed within articles, our meta-analyses reported high levels of heterogeneity. Though this is to be expected due to the high level of variance of delay reported, the study populations used in the meta-analyses were similar in the characteristics of age, proportion of females, two-thirds had defined GCA using a positive temporal artery biopsy (sensitivity analysis showed no difference in delay) and all but two patient samples were from secondary care. Despite this, it should be noted that data included in the meta-analysis did cover a wide time range (1950–2013), in which disease awareness and diagnostic methods will have varied. However, overall, we are confident that our meta-analysis, using reported mean values, provides the best estimate available of diagnostic delay in GCA patients.

## Conclusions

Despite the reported time-period of diagnostic delay being considerably varied within some article samples, on average, patients experience a 9-week delay between the onset of their symptoms and receiving a diagnosis of GCA. Even when the patient has a ‘classical’ cranial presentation, delay remains considerable. In view of the potentially serious consequences of a missed GCA diagnosis, a reduction in diagnostic delay would be beneficial and could result in overall cost savings for healthcare systems [53]. Our research provides a new evidence-based benchmark of diagnostic delay of GCA against which future efforts to reduce this problem can be measured and supports the need for improved public awareness and fast-track diagnostic pathways.

## Additional file

Additional file 1: Table S1. Characteristics of samples not-included in meta-analyses. **Table S2.** Characteristics of articles additionally included for giant cell arteritis (GCA)-specific characteristic analysis. **Table S3.** Article quality appraisal scores using the Newcastle-Ottawa Scale (NOS). **Figure S1.** Meta-analysis of time-periods of delay in receiving a diagnosis of GCA (Original SD only). **Figure S2.** Meta-analysis of time-periods of delay in receiving a diagnosis of GCA (Imputed SD only). **Figure S3.** Meta-analysis of time-periods of delay in receiving a diagnosis of GCA (GCA diagnosis through temporal artery biopsy only). (DOCX 70 kb)

## Abbreviations

ACR: American College of Rheumatology
CI: confidence intervals
GCA: giant cell arteritis
PI: prediction intervals
PMR: polymyalgia rheumatica
RA: rheumatoid arthritis
SD: standard deviation
