# Supplementary material for: Diagnostic delay for giant cell arteritis – a systematic review and meta-analysis
Source: BMC Med. 2017 Jun 28;15:120. doi: 10.1186/s12916-017-0871-z (PMC5488376; doi:10.1186/s12916-017-0871-z)
Supplement: Additional file 1: Table S1. — Characteristics of samples not-included in meta-analyses. Table S2. Characteristics of articles additionally included for giant cell arteritis (GCA)-specific characteristic analysis. Table S3. Article quality appraisal scores using the Newcastle-Ottawa Scale (NOS). Figure S1. Meta-analysis of time-periods of delay in receiving a diagnosis of GCA (Original SD only). Figure S2. Meta-analysis of time-periods of delay in receiving a diagnosis of GCA (Imputed SD only). Figure S3. Meta-analysis of time-periods of delay in receiving a diagnosis of GCA (GCA diagnosis through temporal artery biopsy only). (DOCX 70 kb) [file 12916_2017_871_MOESM1_ESM.docx]

**Additional file 1:**

Table S1: Characteristics of samples not-included in meta-analyses

Table S2: Characteristics of articles additionally included for GCA-specific characteristic analysis

Table S3: Article quality appraisal scores using the Newcastle-Ottawa Scale (NOS)

Figure S1: Meta-analysis of time-periods of delay in receiving a diagnosis of GCA (Original SD only)

Figure S2: Meta-analysis of time-periods of delay in receiving a diagnosis of GCA (Imputed SD only)

Figure S3: Meta-analysis of time-periods of delay in receiving a diagnosis of GCA (GCA diagnosis through TAB only)

Table S1: Characteristics of samples not-included in meta-analyses

|  |  |  | **Gender** |  | **Age** | | |  | **Reported diagnostic delay** | | | |
| --- | --- | --- | --- | --- | --- | --- | --- | --- | --- | --- | --- | --- |
| **Lead author** | **Definition of GCA** | **n** | **% F** |  | **Mean** | **SD** | **Range** |  | **Time** | **Mean** | **SD** | **Range** |
| Calamia | Positive TAB for GCA after fever was initial symptom | 15 | 66.7 |  | 67 | - | 57-75 |  | M | 3* | - | 1-16 |
| Karanjia | Positive TAB for GCA and/or study defined clinical criteria | 63 | - |  | - | - | - |  | D | 52 | - | - |
| Kelkel | Positive TAB for GCA and/or study defined clinical criteria | 130 | 74.6 |  | 76 | 7.5 | 60-92 |  | M | 5* | - | 0.5-48 |
| Hu | Positive TAB for GCA or on clinical grounds (response to steroids) | 16 | 6.3 |  | 43.1 | - | 28-60 |  | M | 5.5 | - | 0.25-24.3 |
| Nuenninghoff | GCA defined using 1990 ACR criteria | 168 | 79.2 |  | 75.6 | - |  |  | D | 40* | - | 21-89 |
| Loddenkemper | Positive TAB for GCA | 90 | 74.4 |  | 74.6 | 7.8 | - |  | D | 125* |  | 2-2555 |

ACR = American College of Rheumatology. Time: D = days, W= weeks & M = months. TAB = Temporal Artery Biopsy. *Reported as median

Table S2: Characteristics of articles additionally included for GCA-specific characteristic analysis

|  |  |  | **Gender** |  | **Age** | | |  | **Reported diagnostic delay** | | |
| --- | --- | --- | --- | --- | --- | --- | --- | --- | --- | --- | --- |
| **Lead author** | **Definition of GCA** | **n** | **% F** |  | **Mean** | **SD** | **Range** |  | **Mean (Weeks)** | **SD** | **Range** |
| Gonzalez-Gay | Positive TAB for GCA with visual manifestations | 42 | 54.8 |  | 75.1 | 6.7 | - |  | 9.6 | 11 | - |
|  | Positive TAB for GCA without visual manifestations | 119 | 47.1 |  | 74.6 | 6.9 | - |  | 11.5 | 13 | - |
| Schmidt | Bilateral blindness from GCA | 5 | 60 |  | 81.6 | - | 76-86 |  | 7 | 3* | 2-14 |
| Gonzalez-Gay | GCA, but negative TAB | 29 | 62.1 |  | 74.3 | 7.5 | - |  | 8 | 4* | 4-20 |
|  | Positive TAB for GCA | 161 | 50.9 |  | 74.8 | 6.8 | - |  | 7 | 1.7* | 4-14 |
| Gonzalez-Gay | Positive TAB for GCA – Men | 97 | - |  | 74.5 | 6.6 | - |  | 9.7 | 13 | - |
|  | Positive TAB for GCA – Female | 113 | 53.8 |  | 74.7 | 7.3 | - |  | 11.0 | 10 | - |
|  | Positive TAB for GCA – Rural | 132 | 46.2 |  | 74.0 | 6.7 | - |  | 9.9 | 12 | - |
|  | Positive TAB for GCA – Urban | 78 | 66.7 |  | 75.6 | 7.3 | - |  | 11.1 | 11 | - |
| Gonzalez-Gay | Positive TAB for GCA – With headache | 203 | 53.2 |  | 74.7 | 6.7 | - |  | 9.2 | 9.9 | - |
|  | Positive TAB for GCA – Without headache | 37 | 59.5 |  | 75.2 | 6.9 | - |  | 16.6 | 15 | - |
|  | Positive TAB for GCA – With PMR | 96 | 60.4 |  | 73.4 | 6.3 | - |  | 13.4 | 12 | - |
|  | Positive TAB for GCA – Without PMR | 144 | 50 |  | 75.6 | 6.9 | - |  | 8.3 | 10 | - |
|  | Subclinical GCA | 18 | 66.7 |  | 75.6 | 5.9 | - |  | 16.3 | 15 | - |
|  | Biopsy-proven GCA | 222 | 53.2 |  | 74.7 | 6.8 | - |  | 9.9 | 11 | - |
| Lopez-Diaz | Positive TAB for GCA - <69 years | 46 | 52.2 |  | 75.1 | 6.8 | - |  | 13.2 | 12.8 |  |
|  | Positive TAB for GCA - ≥70 years | 227 | 53.7 |  |  |  |  |  | 9.4 | 10.2 |  |

ACR = American College of Rheumatology. ESR = Erythrocyte Sedimentation Rate. PMR = Polymyalgia Rheumatica. Time: D = days, W= weeks & M = months.

MRR = Medical Record Review; TAB = Temporal Artery Biopsy. *SD imputed from range

Table S3: Article quality appraisal scores using the Newcastle-Ottawa Scale (NOS)

| **Article** | **Year** | **Selection** | | **Outcome** |
| --- | --- | --- | --- | --- |
|  |  | **1** | **3** | **1** |
|  |  | Is exposed cohort  representative? | How was exposed  cohort selected? | How was  outcome assessed? |
| Calamia | 1981 | B* | A* | B* |
| Bella Cueto* | 1985 | B* | A* | D |
| Karanjia | 1989 | B* | A* | B* |
| Desmet* | 1990 | B* | A* | B* |
| Kelkel | 1991 | B* | A* | B* |
| Myklebust* | 1996 | A* | A* | B* |
| Brack* | 1999 | B* | A* | B* |
| Duhaut* | 1999 | A* | A* | B* |
| Nesher* | 1999 | B* | A* | A |
| Hu | 2002 | B* | A* | D |
| Liozon* | 2003 | B* | A* | B* |
| Nuenninghoff | 2003 | A* | A* | B* |
| Gonzalez-Gay* | 2004 | B* | A* | B* |
| Pease* | 2005 | B* | B* | B* |
| Loddenkemper | 2007 | B* | A* | B* |
| Mari* | 2009 | B* | A* | B* |
| Ezeonyeji* | 2011 | B* | A* | B* |
| Mackie* | 2011 | A* | A* | B* |
| Czihal* | 2012 | B* | A* | B* |
| Prieto-Gonzalez* | 2012 | B* | A* | B* |
| Patil* | 2015 | A* | A* | B* |
| Singh* | 2015 | A* | A* | B* |
| Gonzalez-Gay^ǂ^ | 2000 | A* | A* | B* |
| Schmidt^ǂ^ | 2000 | C | D | D |
| Gonzalez-Gay | 2001 | A* | A* | B* |
| Gonzalez-Gay | 2003 | A* | A* | B* |
| Gonzalez-Gay^ǂ^ | 2005 | A* | A* | B* |
| Lopez-Diaz | 2008 | A* | A* | B* |

*Included in delay meta-analyses. ^ǂ^Included in characteristic-specific delay meta-analysis.

A indicates the highest methodological quality whereas D indicates the worst quality; An asterisk (*) denotes that the article has scored highest for that particular criterion. A comma (,) separating two scores denotes that an article i) matched exposed and non-exposed and ii) adjusted for potential confounding factors

Figure S1: Meta-analysis of time-periods of delay in receiving a diagnosis of GCA (Original SD only)

Mean weeks of delay

(95%CI)

Weight

(%)

Author Year Country n

Figure S2: Meta-analysis of time-periods of delay in receiving a diagnosis of GCA (Imputed SD only)

Mean weeks of delay

(95%CI)

Weight

(%)

Author Year Country n

Figure S3: Meta-analysis of time-periods of delay in receiving a diagnosis of GCA (GCA diagnosis through TAB only)

Mean weeks of delay

(95%CI)

Weight

(%)

Author Year Country n
